# ER homeostasis and autophagy

**DOI:** 10.1042/EBC20170092

**Published:** 2017-12-12

**Authors:** Matthew Smith, Simon Wilkinson

**Affiliations:** Edinburgh Cancer Research UK Centre, MRC Institute of Genetics and Molecular Medicine, University of Edinburgh, Edinburgh EH4 2XR, U.K.

**Keywords:** Autophagy, ER-phagy, ER stress, ER homeostasis, proteostasis, unfolded protein response

## Abstract

The endoplasmic reticulum (ER) is a key site for lipid biosynthesis and folding of nascent transmembrane and secretory proteins. These processes are maintained by careful homeostatic control of the environment within the ER lumen. Signalling sensors within the ER detect perturbations within the lumen (ER stress) and employ downstream signalling cascades that engage effector mechanisms to restore homeostasis. The most studied signalling mechanism that the ER employs is the unfolded protein response (UPR), which is known to increase a number of effector mechanisms, including autophagy. In this chapter, we will discuss the emerging role of autophagy as a UPR effector pathway. We will focus on the recently discovered selective autophagy pathway for ER, ER-phagy, with particular emphasis on the structure and function of known mammalian ER-phagy receptors, namely FAM134B, SEC62, RTN3 and CCPG1. Finally, we conclude with our view of where the future of this field can lead our understanding of the involvement of ER-phagy in ER homeostasis.

## Introduction

The endoplasmic reticulum (ER) is an intracellular organelle that consists of a continuous network of membranous sheets and tubules spanning the cytoplasm. A lipid bilayer segregates the ER lumen from the cytosol. The ER acts as a reservoir for calcium cations (Ca^2+^). These are maintained at a relatively high concentration within the lumen and can be released during cell signalling responses [1]. The other function of the ER is biosynthesis. ER membranes are divided into two conceptual types, rough and smooth ER, present in different proportions and abundances in different cell lineages, although these are interconnected compartments and gradients of function likely exist. The smooth ER is the site for the biosynthesis of lipids and steroid hormones, and acts as a hub for detoxification enzyme activity [2]. An expansive and specialized smooth ER, the sarcoplasmic reticulum, is present in muscle, wherein it acts as the major calcium store for release during contraction. The rough ER is studded with ribosomes that co-translationally insert nascent polypeptide chains encoding transmembrane or secretory proteins. In the lumen, these proteins fold with the assistance of ER-luminal chaperone proteins, which bind, retain and prevent the aggregation of partially folded substrates [3]. Folding is also aided by post-translational modifications such as N-linked glycosylation, mediated via glycosylases, and intramolecular disulphide bond formation and rearrangement, catalysed by protein disulphide isomerases (PDI) [4,5]. Chaperone binding also prevents secretion from the ER of incompletely folded proteins. The high luminal Ca^2+^ concentration facilitates chaperone protein function [4]. A distinct redox potential in the ER lumen—a more oxidizing environment than the cytoplasm—optimizes PDI activity [5].

All cells require ER but some specialist types have a particularly heavy demand for certain ER functions. For example, hepatocytes are a major site of lipid synthesis and have expansive smooth ER. Similarly, plasma cells (effector B cells) and exocrine cells, which secrete abundant immunoglobulins and zymogens respectively, have a high abundance of rough ER [6].

## ER homeostasis

**Figure 1:**
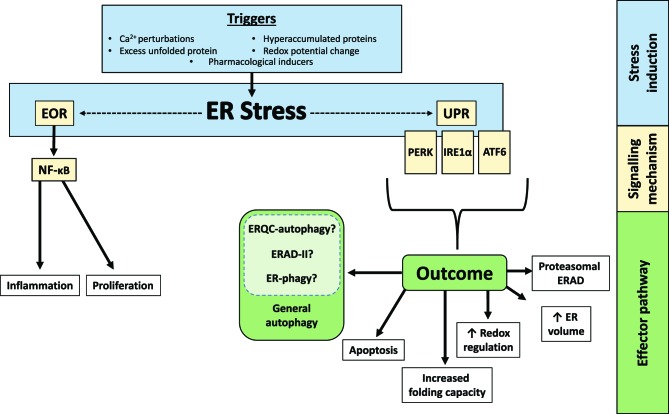
Outcomes of ER stress Perturbation of ER homeostasis or ‘ER stress’ (blue), is ameliorated by the triggering of signalling cascades (yellow), which in turn engage downstream effector mechanisms (green). Generally speaking, these mechanisms restore homeostasis. This review will discuss these pathways and mechanisms, with particular focus on autophagy as a potential effector and, in further detail, selective autophagic degradation of ER luminal contents or ER (ER-quality control (ERQC)-autophagy, ER-associated degradation (ERAD)-II and ER-phagy).

Signalling sensors within the ER detect perturbations within the lumen (ER stress) and employ downstream signalling cascades that engage effector mechanisms to restore homeostasis (Figure 1). These mechanisms include increasing the capacity of the ER, increasing degradation of ER luminal proteins or up-regulating chaperones and luminal protein modification or folding enzymes. This review will focus on ER homeostatic pathways, with a particular emphasis on the emerging role of autophagy as a potential effector mechanism.

Physiologically, ER stress occurs upon, for example, changes to luminal Ca^2+^ concentration, redox status, increased abundance of unfolded proteins and/or hyperaccumulation of proteins. Conditions that produce this include heavy biosynthetic demand, hypoxia, redox stress, deregulated Ca^2+^ homeostasis and crises such as metabolite insufficiency or low intracellular ATP levels. ER stress can also be produced experimentally with drugs that perturb calcium homeostasis, alter redox status or inhibit glycosylation (Figure 1).

Defects in sensing and signalling pathways downstream of ER stress are associated with numerous pathological conditions including diabetes, non-alcoholic fatty-liver disease, Parkinson’s and Alzheimer’s diseases and cancers such as hepatocellular carcinoma [7,8].

Two distinct pathways for response to ER stress have been proposed, comprising the less well-understood ER-overload response (EOR) and the extensively characterized unfolded protein response (UPR). EOR is triggered by hyperaccumulation of ER-resident proteins, not necessarily unfolded protein. Here, the ER releases luminal Ca^2+^, which stimulates reactive oxygen species production that in turn activates NF-κB signalling [9]. Ultimately, NF-κB up-regulates a variety of transcripts that promote proliferation and inflammation. However, little is known about the mechanisms of EOR, particularly if there is any link with autophagy function. It can inhibit viral protein replication, suggesting that it may act as a rapid cellular antiviral response [10,11]. Moreover, EOR activated by Hepatitis C viral proteins leads to an increase in cancer-related gene expression, suggesting a role in hepatocellular carcinoma progression [11,12].

The UPR acts to restore defective proteostasis upon detection of unfolded protein in the ER lumen. It involves up-regulated transcription of redox enzymes, chaperones (such as binding immunoglobulin protein/78-kDa glucose-regulated protein, abbreviated BiP/Grp78), foldases (chaperones catalysing folding via additional enzymatic activity, including PDIs), glycosylases and, additionally, via the transcription factors C/EBP and SREBP1/2, lipid-synthesizing enzymes, which facilitate ER membrane expansion [13,14]. The UPR has three signalling arms, each emanating from an ER integral membrane sensing protein. These sensors are inositol-requiring enzyme 1α (IRE1α), protein kinase RNA-like ER kinase (PERK) and activating transcription factor (ATF) 6. Upon accumulation of misfolded proteins in the ER lumen, BiP/Grp78 is titrated away from the luminal domains of these sensor proteins, releasing them from their monomeric, inactive states and, in the case of ATF6, allowing transit to the Golgi.

IRE1α possesses endoribonuclease activity that splices X-box binding protein 1 (*XBP1*) transcripts, permitting generation of the transcription factor XBP1-S, which up-regulates genes such as ER luminal chaperones and ER-associated degradation (ERAD) machinery component [15]. Up-regulation of chaperones increases the capacity of the ER to deal with unfolded protein. Until recently, ERAD—the removal from the ER of proteins by retrotranslocation into the cytosol and destruction at the proteasome—was thought to be the major mechanism to degrade ER proteins. This process promotes ER homeostasis independently from, but also as an effector of, the UPR, given the transcriptional up-regulation of components of its machinery by the latter [15,16]. ERAD (or ERAD-I) should not be confused with ERAD-II, one of several terms coined recently to describe phenomena where lysosomal, rather than proteasomal, activity appears to result in degradation of ER luminal content (see ‘Autophagy and its role in ER homeostasis’ section). Finally, IRE1α endonuclease activity can also act to degrade secretory protein mRNAs present at the ER in a process termed regulated IRE1α-dependent decay of mRNAs (RIDD), rebalancing protein production and folding capacity [17].

ATF6 differs from other UPR sensors, as it is released from BiP/Grp78 binding to undergo anterograde transport from the ER to the Golgi after ER stress, where it is cleaved by proteases (S1P and S2P, Site 1 and Site 2 proteases) to produce a transcriptionally active polypeptide that can now translocate to the nucleus [18]. Activated ATF6 up-regulates the transcription of ER chaperones, ERAD components and *XBP1* mRNA [19–21]. The latter illustrates that the three arms of the UPR may feedback upon one another.

PERK activation occurs with slower kinetics than ATF6 and IRE1α activation upon acute ER stress [22,23]. PERK is a serine-threonine protein kinase that phosphorylates the translation initiation protein, eukaryotic initiation factor-2α (eIF2α), at Ser^51^, in order to arrest global translation. This results in fewer new protein molecules entering the ER. However, paradoxically, some mRNAs are translationally up-regulated under these conditions, such as ATF4. ATF4 can up-regulate the transcription of C/EBP homologous protein (CHOP) which can then up-regulate growth arrest and DNA damage inducible 34 (GADD34) protein levels. CHOP is a transcription factor that drives pro-apoptotic gene transcription, whereas GADD34 protein dephosphorylates eIF2α, providing negative feedback within the stress response [24,25].

Often, if the UPR fails to resolve ER stress, then activation of cell death by CHOP or other mechanisms such as IRE1α-driven, TRAF2-dependent activation of JNK, and consequent apoptosis, occurs [26]. However, some cell types, both primary and cancerous, are able to withstand constitutive, low-level ER stress and maintain viability, with concomitant chronic up-regulation of UPR transcripts. Some specialized cell types may exhibit UPR-like responses in the differentiated state. Examples include plasma [27,28], osteoblast [29], pancreatic acinar [6], Paneth (intestinal lysozyme granule secreting cells) [30], hepatocyte [31,32] and salivary gland [6] cells, which have an expanded ER relative to their precursor cell state. For example, *XBP1* up-regulation, which in turn generates XBP1-S and leads to the production of molecules required to populate the expanded ER, such as chaperones, is required for plasma cell differentiation [33–35].

## Autophagy and its role in ER homeostasis

One of the more recent effector mechanisms proposed for the UPR is macroautophagy (hereafter autophagy). Autophagy seals off portions of the cytosol within double-membraned vesicles, called autophagosomes. These fuse with lysosomes, wherein their cargo is degraded [36]. The core, conserved elements of this process are covered in depth elsewhere in this book. Here, we will briefly recap two of the core autophagy protein complexes required for understanding of the discussion further on in this chapter. Focal adhesion kinase family kinase-interacting protein 200-kDa (FIP200) is a key autophagy-related (ATG) protein in most forms of autophagy, which assists in nucleation of autophagosomes via scaffolding unc-51-like kinase 1/2 (ULK1/2) kinase activity, which phosphorylates other proteins required for autophagy [37]. It has been known for some time that the ER can platform formation of autophagosomes, potentially at sites of mitochondrial contact [38–40], generating a cradle from which the isolation membrane, the precursor membrane to the autophagosome, extrudes. The isolation membrane is where ‘early’ ATG protein complexes, including those containing FIP200, are concentrated. The subsequent extension of the tubular isolation membrane and self-enclosure results in the distinctive double lipid bilayer structure of the mature autophagosome. Downstream of ULK1 activity, a key protein grouping is ATG8-family members, of which there are six paralogues in mammals. These are microtubule-associated protein 1A/1B-light chain 3 (LC3) A, B and C and γ-aminobutyric acid receptor associated protein (GABARAP), GABARAPL1 and GABARAPL2 [41]. These ubiquitin-like proteins are lipidated and thus covalently attached to nascent autophagosomal membranes.

Autophagy can be a non-selective mechanism that degrades general cytosol. However, autophagy is frequently selective in nature, targeting damaged organelles or aberrant intracellular structures, referred to as ‘cargo’, such as peroxisomes [42], mitochondria [43,44] and lysosomes [45], ubiquitinated protein aggregates and foreign pathogens [38,39,46–48]. A key molecular component of a given selective autophagy pathway is the cargo receptor(s). The canonical form of this receptor class in mammals bridges cargo to ATG8 family protein(s) by binding both simultaneously. ATG8 binding is mediated via linear peptide regions called LC3-interacting region (LIR) motifs [48,49]. A prime example of this is the well-known receptor protein, p62/SQSTM1, which links cargo to ATG8, for example during cytosolic protein aggregate autophagy.

**Figure 2:**
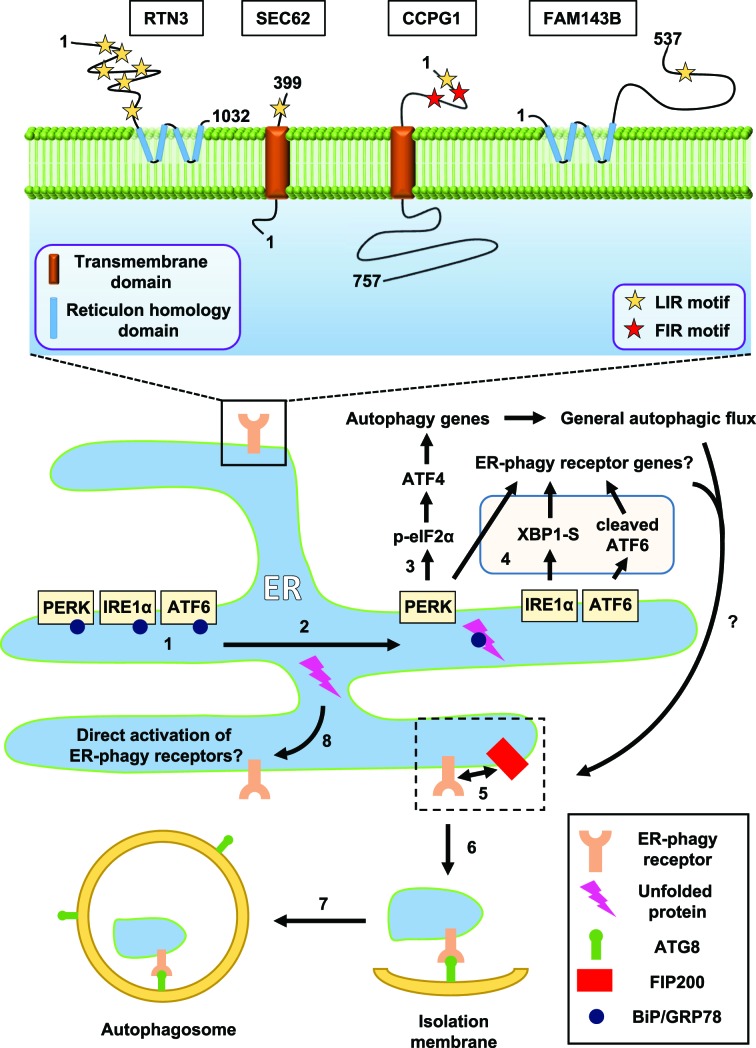
Engagement of autophagy by ER stress and molecular model for ER-phagy events (1) Under normal ER homeostasis, the ER luminal chaperone protein BiP/GRP78 binds to the UPR sensor proteins PERK, IRE1α and ATF6. (2) Upon the presence of ER stress caused by unfolded protein (pink lightning), BiP/GRP78 dissociates from UPR sensors and binds to the unfolded protein, thus activating ER stress sensors. (3) PERK is able to up-regulate the transcription of numerous autophagy genes and cargo receptors through its effector transcription factors ATF4 and CHOP, resulting in an increase in general autophagic flux. (4) Any of the UPR sensors could hypothetically increase the transcription in ER-phagy receptor genes. In the case of *CCPG1*, this indeed occurs. (5) In the case of CCPG1 protein, an interaction with FIP200 is required for recruitment of ER into autophagosomes. It is unclear whether this happens at the ER surface (depicted) or at a latter stage of the pathway. FIP200 is rarely found on the inner surface of autophagosomes, arguing for the former. (6) The ER becomes scissioned. Concomitant with this, ER-phagy receptors bind to ATG8 proteins via their LIR motifs, linking fragmented ER to the isolation membrane. (7) The isolation membrane grows and encloses to form an autophagosome, which will eventually fuse with the lysosome and degrade the ER fragment. (8) It is hypothetically possible that ER stress directly engages and activates ER-phagy receptors independent of transcriptional induction and UPR sensors. To date, there are four described mammalian ER-phagy receptors (top panel). All receptors share the common characteristic of at least one cytosolic LIR motif (yellow star). CCPG1 possesses additional cytosolic FIP200-interacting region (FIR) motifs (red star).

It is becoming evident that ER stress signals can lead to an increase in general autophagy action (Figure 2). Tunicamycin and thapsigargin, two pharmacological inducers of the UPR, drive autophagosome formation in the human neuroblastoma cell line SK-N-SH and increased LC3 lipidation in mouse embryonic fibroblast (MEF) cells, dependent upon the IRE1 sensor [50]. In human glioblastoma and several adenocarcinoma cell lines, it was shown that ER stress downstream of hypoxia results in transcriptional up-regulation of *MAP1LC3B* (*LC3B*) and *ATG5*, via ATF4 and CHOP [51]. Here, the induced autophagy had a prosurvival role. When autophagy was induced by leucine starvation in MEFs, a range of core autophagy genes and cytosolic cargo receptors were identified as PERK-dependent ATF4 transcriptional targets, including *Map1lc3b, Atg5, Atg3, Atg7, Atg10, Atg12, Atg16l1, Becn1, Gabarap, Gabarapl2, p62* and *Nbr1* [52]. Other pharmacologic ER stressors inducing general autophagic flux include A23187, thapsigargin, tunicamycin, brefeldin A (HCT116 human colon and DU145 human prostate carcinoma cells, and MEFs), cocaine (A172 human astrocytoma cells) and 14-deoxy-11,12-didehydroandrographolide (T47D human breast carcinoma cells) [53–55]. The selectivity of autophagy was addressed in HCT116 and DU145 cells via ultrastructural characterization of autophagosomes, which were shown to contain a variety of cargo, suggesting a general up-regulation of relatively non-selective autophagy [53]. Interestingly, in yeast, the specific activity of Atg1, the orthologue of ULK1, is increased upon ER stress, without evident transcriptional induction [56]. This has not yet been observed in mammals.

None of the above studies addressed whether autophagy directly regulates ER homeostasis or showed that autophagy could participate in selective degradation of the ER. However, loss of all autophagy function by knockout of conditional *Atg7* flox alleles in T lymphocytes (Lck-Cre) or *Atg5* flox alleles in B lymphocytes (CD19-Cre) resulted in expanded ER and elevated ER stress signalling, suggesting a role for autophagy in ER homeostasis [57,58]. Additionally, when wild-type yeast were treated with the UPR inducer DTT, ultrastructural analysis showed, for one of the first times, that ER in any organism could be selectively sequestered in autophagic-like vacuoles [59]. These early observations generated the idea that autophagy might both regulate ER function and even do so by direct action on the ER. In the latter instance, three pathways for putative direct regulation of ER homeostasis by selective autophagy are described, with the terms ‘ER-quality control autophagy’ (‘ERQC autophagy’), ERAD-II and ER-phagy proposed. The degree of overlap between these processes is currently unclear. ER-phagy is presently the mechanistically best-described pathway.

ERQC autophagy was used as a term to describe a mammalian process in which disease-associated, conformer mutants of proteins are removed from the lumen by autophagy without large portions of the ER itself being degraded [60,61]. An example of this is the degradation of the mutant form of the G-protein-coupled receptor E90K-GnRHR [61]. There is little mechanistic information on this phenomenon. A potentially similar phenomenon was reported in mammals for removal of insoluble molecules of mutant protein from the ER via a lysosomal route, for which the term ERAD-II was proposed, in analogy to proteasomal ERAD (or ERAD-I) [62].

ER-phagy involves the sequestration of portions of ER cisternae into autophagosomes and occurs in both yeast and mammals (Figure 2). Potential examples include the ER stress associated inhibition of mTOR and incorporation of ER into autophagosomes in *Listeria*-infected phagocytes, which may ameliorate ER stress and death [63]. Indeed, it is presumed that general up-regulation of autophagy capacity, as described above, would translate into a commensurate greater rate of ER-phagy, but this remains unproven. However, recent findings that have given the phenomenon of ER-phagy mechanistic credence and have allowed direct testing of its role in cellular function and pathology, are the discovery of ER-phagy-specific cargoes and cargo receptors, both in yeast [64,65] and mammals [66–69]. We will examine predominantly mammalian ER-phagy receptors below to illustrate their mechanisms of action and explore their potential integration into ER stress responses.

Four main mammalian ER-phagy receptors have been identified *in vitro*, namely FAM134B (U2OS and MEF cells), SEC62 (HeLa and MEF cells), RTN3 (U2OS and MEF cells) and CCPG1 (HeLa and A549 cells) [66–69]. These share some common and divergent principles of action (Table 1). Additionally, two ER-phagy receptors exist in yeast, Atg39 and Atg40 [65], which work along broadly similar principles (Table 1). Other well-known autophagy receptor proteins, such as p62/SQSTM1, may possibly have a role in ER homeostasis, although it is not clear that this is due to any direct function in ER-phagy [70]. In the majority of the above cases, ultrastructural analyses have shown that ER-phagy receptors drive the sequestration of isolated fragments of ER into an autophagosomal lumen defined by discrete, delimiting membrane(s) [65–68].

**Table 1:** Known ER-phagy receptors in yeast and mammals, and their known functions and characteristics

Receptor	General description	AIM/LIR	Other ATG-interaction motifs	Physiological role	Reticulon homology domain (RHD)	Transmembrane protein	Region of ER
**Yeast (*Saccharomyces cerevisiae*)**							
Atg39	Localized to the peripheral ER and nuclear envelope. Also participates in nucleophagy as it encapsulates nuclear contents as well as ER membranes	**W**NL**V**	Atg11BR	Regulates perinuclear ER and nuclear morphology	No	Yes	Perinuclear
Atg40	Localized predominantly at cytoplasmic and cortical ER. Facilitates the loading of ER sheets and tubules into autophagosomes	**Y**DF**M**	Unknown (proposed Atg11 interaction)	Regulates ER morphology	Yes	Yes	Perinuclear, cortical and cytoplasmic
**Mammals (*Homo sapiens*)**							
FAM134B	Promotes the remodelling and scission of ER sheets through its reticulon domain	**F**EL**L**	Unknown	Health of sensory neurons	Yes	No	Sheets
SEC62	Delivers portions of ER into autophagosomes following ER stress in a process termed ‘recovER-phagy’	**F**EM**I**	Unknown	Unknown	No	Yes	Unknown
RTN3	Promotes the remodelling and scission of ER tubules through its reticulon domain	**F**TL**L**	Unknown	Unknown	Yes	No	Tubules
		**Y**SK**V**					
		**F**EV**I**					
		**W**DL**V**					
		**F**EE**L**					
		**Y**DI**L**					
CCPG1	Delivers portions of ER to autophagosomes in response to ER-stress induction	**W**TV**I**	Two FIR motifs	Luminal proteostasis of exocrine pancreas (acinar cells)	No	Yes	Unknown

The bold-underlined residues are key residues required for ATG8/LC3 binding. Abbreviations: AIM, Atg8-interacting motif (yeast equivalent of an LIR); ATG11BR, Atg11-binding region; FIR, FIP200-interacting region.

ER-phagy receptors are basally ER resident proteins, either transmembrane with associated luminal and cytosolic domains or anchored within ER membranes, and in both instances exposing at least one LIR motif to the cytosol (Figure 2). The LIR motif in all instances is required for ER sequestration into autophagosomes [66–69]. RTN3 possesses a total of six LIR motifs, all of which contribute to ATG8 binding. CCPG1 also contains two novel FIP200-interacting region (FIR) motifs [69]. As FIR motifs are required for ER-phagy, FIP200 may also thus have a distinct role in cargo selection in addition to its known role in regulating ULK1 activity (Figure 2).

FAM134B, CCPG1 and RTN3 all act to trim ER content, thus maintaining ER morphology [66–69]. Notably, both FAM134B and RTN3 (and yeast Atg40) share the presence of a reticulon homology domain (RHD), which consists of two hairpin helices that anchor the protein to ER membranes. The presence of the RHD facilitates membrane curvature and potentially mediates ER scission to facilitate ER-phagy [65–67]. RTN3 resides in peripheral ER tubules whereas FAM134B is found on perinuclear ER sheets and these subdomain resident receptors act to drive local ER-phagy [67]. For instance, RTN3 specifically drives tubular ER-phagy. Both FAM134B and RTN3 can drive ER-phagy *in vitro* that is stimulated by the general autophagic stimulus of amino acid starvation. *In vitro*, CCPG1 may also participate in ER-stress driven ER-phagy, after DTT treatment. Finally, Sec62 participates specifically in ER-phagy during recovery from ER stressors, clearing fragments of ER enriched in now redundant ER chaperones [68]. This process is distinguished within the larger set of ER-phagy responses by the term ‘recovER-phagy’. These different mechanistic routes for ER-phagy, targeting distinct regions of the ER and at different stages of the ER stress response, suggest functional specialization of ER-phagy pathways, which may have further relevance *in vivo* where the ER exhibits large differences in form and function between different tissue types (see below).

There is emerging evidence for direct links between homeostatic ER responses and ER-phagy from *in vivo* models. Investigations of the physiological role of autophagy have generally involved ablation of all autophagy function by conditional knockout of core *Atg* genes in various tissues in mice, using tissue-specific, promoter-driven recombinases, typically Cre or tamoxifen-inducible Cre-ERT2. Summarizing, autophagy loss *per se* generally disrupts ER morphology and size, and produces stress responses. In Paneth cells, *Atg7* or *Atg16l* were seen to be required to restrain IRE1α activation after ER stress induced by experimental XBP1 down-regulation. Loss of this action leads to intestinal inflammation [71]. Furthermore, knockout of *Atg5* or *Atg7* in pancreatic acinar cells produces ER dilation, stress, inhibition of secretory protein transcription, cell death and inflammation [72,73], although not all reports agree with these findings [74]. However, it is important to note that the contribution of ER-phagy is not precisely interrogated when core autophagy genes are deleted; ER pathology may be an indirect consequence of damaged mitochondria or protein aggregate accumulation, and consequent effects on bioenergetics and signalling. The construction of ER-phagy receptor mutant mice has begun to allow exploration of this issue. *Fam134b^−/−^* mice exhibit dilated ER and Golgi within peripheral sensory neurons and cell death [66]. Intriguingly, *FAM134B* is mutated in human families with heritable sensory neuropathy and these mutations ablate ER-phagy function [66]. In *Ccpg1* hypomorphic animals, the acinar cells within the exocrine pancreas exhibit distended ER and insoluble ER luminal protein accumulation, as well as elevated UPR [69]. Few other tissues are reported to be affected by loss of ER-phagy receptor function, indicating that physiologically important ER-phagy receptors remain undiscovered or untested. This observation befits the functional specialization of ER within different cell lineages.

## Future directions

In the authors’ view, a major source of outstanding questions in the field of mammalian ER homeostasis centre upon the mechanism and function of ER-phagy.

One question: is whether there is a relationship between ER-phagy and mechanistically opaque processes such as ERQC autophagy and ERAD-II? What degree of overlap in players and mechanisms is there here? Additionally, what molecular determinants mark an ER-phagy receptor, other than LIR motifs? For example, CCPG1 additionally possesses FIR motifs for recognition of the ER by autophagy. Linked to this, it is also crucial to consider whether ER-phagy receptors merely mark ER membrane for degradation or play an active role in scissioning ER membrane to permit sequestration or in sensing ER stress. As a potential example of the former, FAM134B and RTN3 have reticulon domains that might assist ER membrane breakage. In the latter category, some of these receptors have luminal polypeptide regions and it might be conjectured that they could directly sense changes in redox, luminal [Ca^2+^] or unfolded protein in parallel with canonical sensors. In the case of CCPG1, the ER luminal domain contains a coiled-coil forming region, and it is possible that regulated multimerization/clustering could modify function, as suggested for RTN3 [67,69]. Cytosolic domains might also play a role within the downstream relays of ER stress signalling pathways. For example, some cargo receptors acting in other selective autophagy paradigms bear phosphoregulable LIR motifs [75,76].

Does ER stress specifically trigger ER-phagy, non-selective autophagy or a combination of both, and in which tissue types and to what ultimate purpose? The finding that the UPR can transcriptionally up-regulate *CCPG1* provides a mechanistic link between ER stress and selective autophagy of the ER. The *Ccpg1* promoter also binds the transcription factor MIST1 [77], which is predominantly expressed in professional secretory cells such as pancreatic acinar cells. MIST1 expression is required for correct differentiation here and is itself dependent upon the IRE1α-XBP1 arm of the UPR [78,79], providing a potential tissue-specific link between CCPG1 up-regulation and its role in ER homeostasis. It is therefore of key importance to dissect whether ER stress response pathways can regulate the activity of other ER-phagy receptors, either to activate them acutely or as is likely for CCPG1 *in vivo*, also in part to mediate their tissue-specific expression.

It is difficult to say with exactitude how ER-phagy mediates ER homeostasis at a detailed mechanistic level. It is possible that ER-phagy acts to remodel and rebalance the different regions of the ER to meet fluctuating biosynthetic demand. Alternatively, perhaps proteostatic defects result in localized accumulation of unfolded or aggregated protein, by transport mechanisms within the ER lumen, such that specific portions of ER are ‘sacrificed’ in a piecemeal manner in order to maintain unfolded protein at a manageable level within the lumen. There is also little information on rough ER compared with smooth ER as a cargo of autophagy. Different pathways and different receptors may participate, again pointing to likely tissue-specific divergence of these mechanisms *in vivo*.

Finally, another potentially ER-phagy related mechanism that specialist cells might use to respond to ER stress is secretory autophagy. Paneth cells secrete ER luminal lysozyme via the ER to defend against pathogens. Here, ER stress, triggered by bacterial invasion, results in an increase in secretory autophagy of lysozyme, i.e. the use of the autophagy machinery to build secretory vesicles containing the lysozyme, rather than utilization of the default secretory pathway via the Golgi [80]. Investigations are required to determine if ER-phagy receptors play a role here.

## Conclusion

The UPR and other ER stress response signalling pathways engage multiple effector pathways. A recently emerging effector is autophagy and, of particular interest, ER-phagy. Taken together, the evidence points to a pivotal role for ER-phagy in normal ER homeostasis and overall cell health, particularly in specialized tissue types *in vivo*. Crucially, ER-phagy helps ameliorate the effects of ER stress through the degradation of ER membranes, removal of ER luminal protein aggregates and/or removal of ER-chaperone proteins. Furthermore, ER-phagy also acts to trim ER content, helping maintain the dynamism that is characteristic of this key organelle. Future work will surely expand the molecular components and role of this pathway in currently known and new tissue types.

## Summary

- ER is the key site for the folding of nascent transmembrane and secretory proteins.
- The lumen of ER has a specialized environment to ensure the fidelity of this process.
- Signalling sensors within the ER lumen detect stress and employ downstream cascades to engage effector mechanisms and restore homeostasis.
- The major signalling cascade employed by the ER is the UPR, which translationally and transcriptionally engages a variety of effector mechanisms.
- Selective degradation of the ER by autophagy occurs via a process termed as ER-phagy.
- There are currently four known mammalian ER-phagy receptors; FAM134B, SEC62, RTN3 and CCPG1.
- ER-phagy receptors possess LIR motifs, allowing interactions with ATG8 family proteins.
- ER-phagy has important roles in the physiology of secretory cells *in vivo*.
- General autophagic flux and direct ER-phagy might both be transcriptionally up-regulated by the UPR.

## Abbreviations

ATF: activating transcription factor
ATG: autophagy-related
Bip/Grp78: binding immunoglobulin protein/78-kDa glucose-regulated protein
CHOP: C/EBP homologous protein
eIF2α: eukaryotic initiation factor-2α
EOR: ER-overload response
ER: endoplasmic reticulum
ERAD: ER-associated degradation
ERQC: ER quality control
FIR: FIP200-interacting region
GADD34: growth arrest and DNA damage inducible 34
IRE1α: inositol-requiring enzyme 1α
LC3: light chain 3
LIR: LC3-interacting region
MEF: mouse embryonic fibroblast
PDI: protein disulphide isomerase
PERK: protein kinase RNA-like ER kinase
RHD: reticulon homology domain
UPR: unfolded protein response
XBP1: X-box binding protein 1
